# Prevalence of Patients Who Return to Theatre Post-Adenoidectomy: A Review of Hospital Episode Statistics Data (2012-2019)

**DOI:** 10.7759/cureus.13980

**Published:** 2021-03-18

**Authors:** Navdeep Bhamra, Max S Osborne, Edward Balai, Karan Jolly, James Barraclough

**Affiliations:** 1 Otolaryngology, The Royal Wolverhampton Hospitals NHS Trust, Wolverhampton, GBR

**Keywords:** adenoidectomy, post-operative, haemorrhage, adenoid

## Abstract

Introduction

Risk of surgical intervention for post-adenoidectomy haemorrhage can be assessed with the analysis of the Hospital Episode Statistics (HES) data.

Materials and methods

HES data for England from 2012 to 2019 were analysed comparing the coded number of adenoidectomy procedures to the number of surgical arrests of post-adenoidectomy haemorrhage in adolescents/adults and children.

Results

Between April 2012 and April 2019, of 47,597 procedures, 52 (0.11%) patients required surgical arrest of post-adenoidectomy haemorrhage. In adults (n = 5,379), 11 patients returned to theatre for control of post-operative bleeding, whereas 41 children (n = 42,218) required this intervention. The total number of adenoidectomies was 3.7 times higher in children; however, adults were statistically two times more likely to require further surgical intervention for arrest of post-adenoidectomy haemorrhage (two-tailed p-value = 0.0031).

Conclusion

Children are more likely to return to theatre for surgical arrest of post-adenoidectomy haemorrhage, with p-values indicating the difference between the incidence of adults and children returning to theatre to be very statistically significant.

## Introduction

Hospital Episode Statistics (HES) is a database containing details of all admissions, Accident and Emergency attendances and outpatient appointments at the National Health Service (NHS) hospitals in England as well as clinical information about diagnoses and operations [1]. HES data cover all private patients treated in NHS hospitals, patients who reside outside of England and care which is delivered by treatment centres (including those in the independent sector) funded by the NHS [1]. Information provided in the database does not extend to specifics regarding patient admission; however, it provides an indication of current surgical rates within English NHS hospitals. HES data on adenoidectomies are available on ‘Main procedures and interventions,’ which is divided into six four-character operative codes (OPCS-4) along with their associated data. The accuracy of these data relies on the correct coding of these operative procedures.

## Materials and methods

The HES data available for ‘Main procedures and interventions’ for the years 2012-2019 were downloaded in Microsoft Excel format. The operative (OPCS-4) codes E20.1, E20.2, E20.4 relating to procedures of the adenoids and code E20.3 relating to the surgical arrest of post-operative bleeding of adenoid were considered. Codes regarding other specified operations on adenoid (E20.8) and unspecified operations on adenoid (E20.9) were excluded from this study. No OPCS-4 code exists for adenotonsillectomy as a single procedure.

The data were compared for children (0-15 years) and adolescents/adults (16+) years and statistically assessed using a chi-square test. This is the same methodology used in a previous study looking at the surgical arrest of post-tonsillectomy haemorrhage [2]. The HES data provided data into 24 age categories.

## Results

Between April 2012 and April 2019, 47,597 adenoidectomies were performed, of which 52 (0.11%) returned to theatre for surgical arrest of post-operative bleeding (Table 1).

**Table 1:** All HES data used in this article. HES, Hospital Episode Statistics

Years	Adenoidectomy (n)			Surgical arrest of post-adenoidectomy haemorrhage (n)			Return to theatre (%)		
	0-15 years	16+ years	Total	0-15 years	16+ years	Total	0-15 years	16+ years	Total
2012-13	5,613	691	6,304	7	1	8	0.12%	0.14%	0.13%
2013-14	6,309	731	7,040	8	1	9	0.13%	0.14%	0.13%
2014-15	6,731	715	7,446	11	1	12	0.16%	0.14%	0.16%
2015-16	6,467	764	7,231	4	3	7	0.06%	0.39%	0.10%
2016-17	6,245	817	7,062	4	1	5	0.06%	0.12%	0.07%
2017-18	5,579	849	6,428	3	1	4	0.05%	0.12%	0.06%
2018-19	5,274	812	6,086	4	3	7	0.08%	0.37%	0.12%
Total	42,218	5,379	47,597	41	11	52	0.10%	0.20%	0.11%

**Figure 1:**
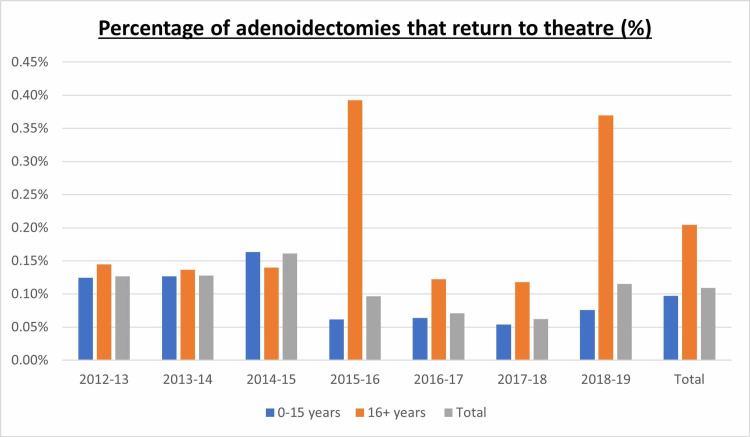
Chart representing the percentage of adenoidectomies that returned to theatre in both adults and children.

In children, 42,218 adenoidectomies were performed and 41 (0.10%) returned to theatre, with the two-tailed p-value of less than 0.0001 indicating that this is very statistically significant. In adolescents/adults, 5,379 adenoidectomies were performed and 11 (0.20%) returned to theatre (Figure 1), with the two-tailed p-value of less than 0.0001 indicating this is very statistically significant. Adolescents/adults were therefore two times more likely than children to return to theatre for control of post-operative bleeding. The difference between the incidence of adolescents/adults and children returning to theatre is very statistically significant (two-tailed p-value = 0.0031).

## Discussion

Post-adenoidectomy haemorrhage is a rare occurrence and less common than the same complication following a tonsillectomy. It is encouraging to see that an overall haemorrhage rate post-adenoid procedure is 0.11%. In a previous study performed looking at 7,946 adenoidectomies performed between 1995 and 2014, only seven (0.09%) patients experienced this post-operative complication [3].

Children had undergone 7.8 times more adenoid procedures than adults, and our results demonstrate that adults are two times more likely to require an arrest of post-adenoidectomy haemorrhage. These findings are in consensus with previous reviews of HES data with regard to tonsil haemorrhage arrest rates, which demonstrated that adults are more likely to require surgery following tonsillectomy than children and that overall number of these procedures had almost doubled since in the instruction of the SIGN (Scottish Intercollegiate Guideline Network) guidelines [2].

The cause for the higher levels of post-operative adenoidal bleeding frequency in adults and for peaks in the 16+ age group in 2015-16 and 2018-19 is difficult to ascertain as individual patient data are not available through HES. Though there is a significant amount of data available through the HES database, one drawback is that the data are macro and not granular. For this reason, it can be considered not to have sufficient depth in order to form a valid analysis. Anecdotally from clinical experience, it can be hypothesised that the indications for adult adenoid procedures and possible temporal effects of recurrent inflammation or infection in adults could result in increased scarring and therefore a more challenging dissection.

Surgical technique may also have an impact on bleeding rates as cold adenoidectomies that use either a curette or an adenotome have been found to have a high incidence of primary bleeding within the first 24 hours of surgery [4]. Hot adenoidectomy techniques such as monopolar and bipolar cautery dissect the tissue with thermal energy (approximately 400°C), have a shorter operative times and have less operative bleeding compared to cold techniques [3]. Longer operating times as with cold techniques are found to be associated clinically and statistically with primary post-operative haemorrhage [5]. Addressing both techniques, it is also noted that ‘hot’ techniques involve either direct (with a dental mirror) or endoscopic visualisation upon performing the procedure [6] which can lead to a more efficient dissection, thus potentially reducing the post-operative haemorrhage rate. Further sub-group analysis of surgical technique (hot vs. cold techniques) in those who returned to theatre for arrest of haemorrhage is unfortunately not available as per the HES database.

With regard to the management of those who require management of post-adenoidectomy haemorrhage, there are recommendations to consider either re-curettage, cauterisation or posterior nasal packing [7-9]. Unfortunately, the only data within the HES database surrounding those who require surgical arrest of haemorrhage and those who bled and were treated by other means are not accounted for within this database.

## Conclusions

We hope that the data presented in this study will help guide clinicians during consenting their patient by providing up-to-date national risk of post-operative haemorrhage in adenoid procedures. It is important to note that HES data is reliant on accurate clinical coding and that true rates of post-operative bleeding may in fact be higher than demonstrated.
